# Gap Junctions Are Involved in the Rescue of CFTR-Dependent Chloride Efflux by Amniotic Mesenchymal Stem Cells in Coculture with Cystic Fibrosis CFBE41o- Cells

**DOI:** 10.1155/2018/1203717

**Published:** 2018-01-11

**Authors:** Annalucia Carbone, Roberto Zefferino, Elisa Beccia, Valeria Casavola, Stefano Castellani, Sante Di Gioia, Valentina Giannone, Manuela Seia, Antonella Angiolillo, Carla Colombo, Maria Favia, Massimo Conese

**Affiliations:** ^1^Division of Internal Medicine and Chronobiology Unit, IRCCS “Casa Sollievo della Sofferenza”, San Giovanni Rotondo, Foggia, Italy; ^2^Department of Medical and Surgical Sciences, University of Foggia, Foggia, Italy; ^3^Department of Medicine and Health Sciences “V. Tiberio”, University of Molise, Campobasso, Italy; ^4^Department of Bioscience, Biotechnology and Biopharmaceutics, University of Bari, Bari, Italy; ^5^Medical Genetics Laboratory, Fondazione IRCCS Cà Granda Ospedale Maggiore Policlinico, Milan, Italy; ^6^Cystic Fibrosis Center, Fondazione IRCCS Cà Granda Ospedale Maggiore Policlinico, Department of Pathophysiology and Transplantation, University of Milan, Milan, Italy

## Abstract

We previously found that human amniotic mesenchymal stem cells (hAMSCs) in coculture with CF immortalised airway epithelial cells (CFBE41o- line, CFBE) on Transwell® filters acquired an epithelial phenotype and led to the expression of a mature and functional CFTR protein. In order to explore the role of gap junction- (GJ-) mediated intercellular communication (GJIC) in this rescue, cocultures (hAMSC : CFBE, 1 : 5 ratio) were studied for the formation of GJIC, before and after silencing connexin 43 (Cx43), a major component of GJs. Functional GJs in cocultures were inhibited when the expression of the Cx43 protein was downregulated. Transfection of cocultures with siRNA against Cx43 resulted in the absence of specific CFTR signal on the apical membrane and reduction in the mature form of CFTR (band C), and in parallel, the CFTR-dependent chloride channel activity was significantly decreased. Cx43 downregulation determined also a decrease in transepithelial resistance and an increase in paracellular permeability as compared with control cocultures, implying that GJIC may regulate CFTR expression and function that in turn modulate airway epithelium tightness. These results indicate that GJIC is involved in the correction of CFTR chloride channel activity upon the acquisition of an epithelial phenotype by hAMSCs in coculture with CF cells.

## 1. Introduction

Respiratory disease is the major cause of morbidity and mortality for cystic fibrosis (CF) patients, who on average survive up to 40 years of age. CF is an autosomal recessive disease, caused by genetic defects in the *CF transmembrane conductance regulator* (*CFTR*) gene on the long arm of chromosome 7. More than 2000 mutations in this gene have been described to date (http://www.genet.sickkids.on.ca/app, last access June 20, 2017), although the deletion of phenylalanine at position 508 (F508del) represents the most frequent mutation, being present in 70% of CF chromosomes worldwide [1]. The F508del CFTR protein is defective in its processing at the level of the endoplasmic reticulum and is directed to the degradation in the proteasome resulting in very low levels of the protein at the apical plasma membrane of airway epithelial cells. The lack of CFTR at the membrane region impairs not only chloride efflux in the airway lumen but also the absorption of sodium ions by epithelial cells, since wild-type CFTR exerts a negative tonic effect on the epithelial sodium (ENaC) channel [2]. Therefore, lack/dysfunction of CFTR eventually leads to dehydration of airway secretions, ablation of the mucociliary clearance, and airway colonisation and infection by opportunistic bacterial pathogens, including the gram-negative *Pseudomonas aeruginosa* [3]. Although this pathophysiologic process is the most accepted model, with a notable exception in the CF pigs (where the basic defect involves the regulation of bicarbonate secretion and the pH of airway secretions) [4], nevertheless, other defects in airway epithelial cells have been described, involving the actin cytoskeleton and the tightness of the epithelium. NHERF1, ezrin, and protein kinase A form a multiprotein complex which tethers CFTR on the apical plasma membrane of airway epithelial cells and guarantees its correct functioning as a chloride channel [5]. In CF cells, homozygous for F508del CFTR, this complex is disrupted and CFTR delocalisation and degradation are associated with disorganization of actin cytoskeleton and tight junction leakiness [6–8].

CF, particularly its lung manifestations, at the moment has no cure. The increase in the median age of survival for CF patients observed in recent years is due to the improvements in chest physiotherapy and antibiotic regimens [9]. New perspectives are offered by the introduction of drugs which can correct the F508del processing defect and potentiate its channel activity [10]. The corrector lumacaftor, the first introduced in the clinics, exerts a limited efficacy on F508del homozygous patients at the lung function level [11].

Stem cell-based therapies could have the advantage of replenishing the niche of the damaged airway epithelium and allow a long-term correction of the underlying basic defects, irrespective of the mutation. Among the possible sources of stem cells for the treatment of lung diseases, embryonic stem cells (ESCs) and induced-pluripotent stem cells (iPSCs) hold interesting properties for their capacity to give rise to a completely differentiated airway epithelium and therefore are useful for airway mucosal repair [12]; however, their employment is limited by concerns regarding tumor formation (both ESCs and iPSCs) and immune response (ESCs). Mesenchymal stem cells (MSCs) are derived from adult tissue and have been evaluated as a potential cell-based therapy for lung diseases [13], including CF [14]. MSCs derived from the amniotic membrane (AMSCs) are considered as a novel cell source for cell transplantation and regenerative medicine [15]. Human AMSCs (hAMSCs) have gained particular attention in this context also because they are obtained from a discarded material after delivery (i.e., the placenta) and have been used as an amniotic membrane in the clinical setting for more than 100 years [16]. hAMSCs have been shown to have beneficial effects when administered in animal models for a large number of diseases, including lung injury [17] and pulmonary fibrosis [18, 19]. We have previously shown that hAMSCs display the ability to differentiate into airway epithelial cells and determine an increase in CFTR maturation and CFTR-dependent chloride efflux in cocultures with immortalised CF bronchial epithelial cells (CFBE41o- line) [20]. Since the correction was not achieved when hAMSCs were cultured separately from CFBE41o- (CFBE) cells, we hypothesised that gap junction (GJ) intercellular communication (GJIC) is likely to have a role in the rescue of basic defects in the CF airway epithelium.

GJs are important for cell-to-cell communication in different processes such as cell morphogenesis, proliferation, and differentiation. They allow the exchange between cells of low molecular weight metabolites, like amino acids, glucose, nucleotides, and second messengers, such as calcium ions, cAMP, ATP, IP3, and small RNAs [21]. GJs are made by docking of two hexameric hemichannels (connexons) of two apposed cells, thus composed of 12 proteins called connexins. More than 20 members have been identified with a wide variability in their tissue distribution [22]. Connexin 43 (Cx43) is ubiquitously expressed in normal tissue of the airways [23, 24] and has been one of the most studied connexins in the functional and immunological evaluation of airway epithelial cells [25–28]. As concerning CF, it has been previously shown that Cx43-mediated GJ intercellular communication (GJIC) coordinates a signaling network to activate CFTR and modulate airway surface liquid volume in Calu-3 cells [27]. Moreover, Cx43 expression is induced by the PAO1 strain of *P. aeruginosa* leading to a regulated increase in GJIC [26], likely being part of the innate immune response orchestrated by airway epithelial cells.

In this study, we have investigated the role of Cx43 and GJIC in the rescue of CFTR-dependent chloride efflux in coculture of CFBE cells with hAMSCs. Downregulation of Cx43 by siRNA transfection inhibited the formation of GJs and ablated the recovery of chloride efflux as well as the increment in transepithelial resistance. Overall, the data strongly indicate that the resumption of CFTR functional expression in CFBE : hAMSCs cocultures is mediated by GJIC.

## 2. Materials and Methods

### 2.1. Cell Cultures

hAMSCs were isolated from term placenta and grown in advanced DMEM supplemented with 10% FBS (heat-inactivated foetal bovine serum), 55 *μ*M *β*-mercaptoethanol, 1% L-glutamine, 1% penicillin/streptomycin (all purchased from Thermo Fisher Scientific, Milan, Italy), and 10 ng/ml epidermal growth factor (EGF) (Sigma-Aldrich, Milan, Italy) [20]. Overall, in this study, we used six hAMSC isolates: three for analysis of Cx43 mRNA/protein expression and GJIC and another three for analysis of CFTR protein expression/function and paracellular permeability. Human immortalised bronchial epithelial cell lines were 16HBE14o- (16HBE), expressing wild-type CFTR, and CFBE41o- (CFBE), homozygous for the F508del allele [29]. Cells were grown at 37°C under 5% CO_2_ on flasks in MEM medium containing 10% FBS, 1% L-glutamine, and 1% penicillin/streptomycin.

Polarised cocultures were obtained by mixing hAMSCs at passage two with CFBE cells at ratio 1 : 5 (2 × 10^4^ hAMSCs mixed with 8 × 10^4^ CFBE cells). Mixed cells were seeded on 6.5 mm diameter Transwell, 0.4 *μ*m pore size (Corning, Acton, MA, USA) at 1 × 10^5^ per filter coated with a solution of 10 *μ*g/ml fibronectin (BD Biosciences, San Jose, CA, USA), 100 *μ*g/ml albumin from bovine serum (Sigma-Aldrich), and 30 *μ*g/ml bovine collagen type I (BD Biosciences) dissolved in MEM. As controls, hAMSCs and epithelial cells were seeded at 2.5 × 10^4^ and 1 × 10^5^, respectively, per filter. Either single cultures or cocultures were analysed when confluent monolayers were obtained, for example, at 6 days. In some experiments, 1 × 10^5^ mixed cells were seeded onto either 24-well plates or glass slides positioned into 10 cm Petri dishes; confluency was obtained also under these experimental conditions.

### 2.2. Evaluation of Transfection Efficiency of Small Interfering RNA

Cocultures were investigated for their capability to uptake small interfering RNA (siRNA) by transfecting them with Quasar 570-conjugated hGAPDH siRNA (Riboxx GmbH, Radebeul, Germany) complexed with polyethylenimine (PEI 25 kDa; Sigma-Aldrich), to enhance the siRNA delivery. Complexes were prepared by mixing 10 nM PEI to 30 nM hGAPDH siRNA (*N*/*P* = 7) and then incubated for 30 min at room temperature. The complexes were added with 200 *μ*l of Opti-MEM (Thermo Fisher Scientific) and were distributed to each coculture mixing before seeding 1 × 10^5^ mixed cells onto 24-well plates.

At 24 h posttransfection, cells in cocultures, treated or not treated with trypan blue 0.04% in PBS in order to quench extracellular fluorescence, were detached with trypsin/ethylenediaminetetraacetic acid (EDTA) and finally resuspended in 50 *μ*l PBS. Cells were analysed by flow cytometry with the FlowSight® IS100 Flow Cytometer (Amnis; Merck Millipore). Brightfield aspect ratio versus brightfield area plots were generated to identify single cell events. The percentage of positive cells was determined after setting the gating on 99% of an untransfected control population of cells and by subtracting the fluorescence of these cells. Ten thousand cells were examined for each sample by using Amnis IDEAS software. Cells were excited with a 488 nm laser light and the percentage of positive cells (emission at 575 nm) was detected.

### 2.3. Downregulation of Cx43 mRNA and Protein

To obtain the downregulation of Cx43 mRNA and protein, polarised cocultures were treated by transfecting active siRNA pool directed against Cx43 (Riboxx) complexed with 10 nM PEI 25 KDa at *N*/*P* = 7, as described above. In the same experiments, untransfected and cocultures transfected with scrambled negative control siRNA (Riboxx) were included. Finally, the complexes were distributed to each coculture, before seeding 1 × 10^5^ mixed cells onto Transwell filters.

### 2.4. Real-Time Quantitative PCR

Cx43 mRNA expression was investigated by real-time PCR analysis. Total RNA was isolated from cells grown under different conditions by using TRIzol™ Reagent (Thermo Fisher Scientific), according to the manufacturer's protocol. One microgram of RNA was reverse transcribed into first strand cDNA with the High-Capacity cDNA Reverse Transcription kit (Thermo Fisher Scientific) by using random primers following manufacturer's instructions.

To evaluate the Cx43 expression, a SYBR® Green-based real-time PCR assay using the comparative method (on the Mx3005P Stratagene instrument, La Jolla, CA, USA) was carried out. PCRs were set up in triplicate in a total volume of 25 *μ*l per capillary. One reaction mixture contained 12.5 *μ*l of Brilliant SYBR Green QPCR Master Mix from Stratagene, including a SureStart Taq DNA polymerase, a dNTP mixture and SYBR Green, MgCl_2_ (2.5 mM), 0.1 *μ*l of forward and reverse primers each (final concentration: 200 nM), 0.375 *μ*l of diluted reference dye (final concentration: 30 nM), 1 *μ*l cDNA, and 10.925 *μ*l H_2_O. *β*-Actin was used as endogenous control (normalizer). The cycling conditions were as follows: an initial activation step of 95°C for 10 min followed by 40 cycles of denaturation of 95°C for 30 sec, annealing at 57°C for 1 min and extension at 72°C for 30 sec. Primer sequences for amplification were Cx43 forward TCGGGTTAAGGGAAAGAG and Cx43 reverse GCTCACTTGCTTGCTTGT.

### 2.5. Evaluation of Gap Junctional Intercellular Communication (GJIC)

Levels of GJIC in cocultures, untransfected and transfected with active or scrambled negative control siRNA, were determined by microinjection of fluorescent Lucifer Yellow (LY) dye into cells and by evaluation of dye spread to adjacent cells [30]. Mixed hAMSC : CFBE cells were treated with either Cx43 siRNA or scrambled siRNA and then seeded onto glass slides. As a control, mercury (II) chloride (Honeywell Fluka™, Thermo Fisher Scientific) was added to 5-day cocultures at the final concentration of 10 nM. After 6 days, five cells per coculture were microinjected with a solution of 5 mg/ml of the membrane-permeant dye LY (Molecular Probes, Eugene, OR, USA) together with 0.5 *μ*g/ml TRITC-dextran 10 kDa (Molecular Probes) by the micromanipulator 5171 and the transinjector 5246 (Eppendorf, Germany). Ten minutes after injection, cells were washed with PBS and fixed with 4% paraformaldehyde (PFA). The glass slide was mounted onto a coverslip in the presence of a drop of VECTASHIELD Antifade Mounting Medium with DAPI (Vector Laboratories, Burlingame, CA, USA). In other experiments, mixed cells were transfected or left untreated and seeded on Transwell for 6 days and then evaluated for LY diffusion. The fluorescence was imaged by a Nikon IntensilightC-HGFI (Nikon, Florence, Italy) through a 60x oil immersion objective. The microscope was equipped with a DAPI filter (358 nm excitation; 461 nm emission), FITC filter (490 nm excitation; 525 nm emission), and a TRITC filter (532 nm excitation; 570 nm emission). Digital images were processed using the NISE elements program (Nikon).

### 2.6. Immunofluorescence and Confocal Analysis

Polarised cells or cocultures were washed three times with PBS and incubated in 2% BSA/PBS for 30 min on ice. Cells were incubated with an anti-CFTR (CF3) mouse monoclonal antibody (dilution 1 : 500; Abcam) followed by a goat anti-mouse IgG conjugated to Alexa Fluor 488 (dilution 1 : 1000; Invitrogen) used as secondary antibody or with an anti-Cx43 (CX-1B1) mouse monoclonal antibody and Alexa Fluor 488 conjugated (dilution 1 : 300; Abcam) in 0.2% BSA/PBS for 1 h on ice. After two washes in PBS, cells were fixed in 3% PFA and 2% sucrose for 10 min. After three washes in PBS, filters were excised and placed side up on a glass slide and overlaid with a drop of Mowiol (Calbiochem, San Diego, CA, USA) followed by a coverslip. Cells were analysed by using a Nikon Eclipse Ti-E C2 confocal microscope. Specimens were viewed through a 60x oil immersion objective, with a 2.5 zooming in some analyses. Digital images were processed by using the program NIS Element (Nikon). Immunofluorescence and confocal analysis on CFTR were performed as established by us in a previous work on CFBE cells [31].

### 2.7. Fluorescence Measurements of Apical CFTR-Dependent Chloride Efflux

Chloride efflux was measured by using the Cl^−^-sensitive dye, N-(Ethoxy-carbonylmethyl)-6-methoxyquinolinium bromide (MQAE) as we previously reported [20]. Briefly, after 6 days of coculture on permeable filter inserts, cells were loaded overnight in culture medium containing 5 mM MQAE at 37°C in a CO_2_ incubator and then inserted into a perfusion chamber that allowed independent perfusion of apical and basolateral cell surfaces. Fluorescence was recorded with a Cary Eclipse spectrofluorometer (Varian). To measure chloride efflux rate across the apical membrane, the apical perfusion medium was changed with a medium in which chloride was substituted with isoosmotic nitrate. All experiments were performed at 37°C in HEPES-buffered bicarbonate-free media (Cl^−^medium: 135 mM NaCl, 3 mM KCl, 1.8 mM CaCl_2_, 0.8 mM MgSO_4_, 20 mM HEPES, 1 mM KH_2_PO_4_, 11 mM glucose, and Cl^−^ free medium: 135 mM NaNO_3_, 3 mM KNO_3_, 0.8 mM MgSO_4_, 1 mM KH_2_PO_4_, 20 mM HEPES, 5 mM Ca(NO_3_)_2_, and 11 mM glucose). CFTR-dependent chloride secretion was calculated as the difference in the rate of change of forskolin-plus 3-isobutyl-1-methylxanthine- (IBMX-) stimulated fluorescence in the absence or presence of apical treatment with the specific CFTR inhibitor, CFTRinh-172 [20]. This technique has been already used by us in the study of the relationship between CFTR activity as a chloride channel and cytoskeletal organization in airway epithelial cells [31].

### 2.8. Western Blotting Analysis

After 16HBE cells, CFBE cells and hAMSC : CFBE cocultures in various conditions (i.e., untransfected and transfected) were cultured for 6 days on Transwells, monolayers were washed with PBS, homogenized in lysis buffer (110 mM NaCl, 50 mM Tris, Triton X-100 0.5%, and Igepal CA-630 0.5%, pH 8.0, with added protease inhibitor mixture), sonicated for 10 sec, and centrifuged for 10 min (16,000 ×g), and then the pellet was discarded. Supernatant protein concentration was measured, and an aliquot of 30 *μ*g of protein was diluted in Laemmli buffer, heated at 100°C for 5 min and separated by 3–8% Tris-acetate gel (Bio-Rad). The gel was transferred to polyvinylidene difluoride membranes (GE Healthcare Italia, Milan, Italy) and processed for Western blotting by using monoclonal CFTR antibody (R&D Systems, MAB25031; dilution 1 : 500), monoclonal Cx43 antibody (CX-1B1; dilution 1 : 500; Abcam), or monoclonal *β*-tubulin (dilution 1 : 1000; Sigma-Aldrich). The secondary antibody was anti-mouse IgG for all primary antibodies (Sigma-Aldrich). Immunocomplexes were detected with LumiGLO reagent (Cell Signaling, EuroClone, Milan, Italy), and densitometric quantification and image processing were carried out by using Adobe Photoshop and the Image software package (version 1.61, National Institutes of Health, Bethesda, MD, USA).

### 2.9. Transepithelial Resistance

TER was measured at day 6, using chopstick electrodes and a volt-ohm meter (Millicell® ERS, Millipore).

### 2.10. Paracellular Permeability

CFBE and CFBE : hAMSC cocultures, grown for 6 days, were evaluated for paracellular permeability by adding a FITC-conjugated dextran (10 kDa [10 s]; Sigma-Aldrich) to the apical side of monolayers. After 20, 40, and 60 min, the apparent permeability (pAPP) was calculated by measuring the fluorescence in the basal medium, as previously described [8].

### 2.11. Apoptosis and Necrosis

CFBE cell monolayers and cocultures at day 6 were evaluated for apoptosis/necrosis rate by using the FlowCellect™ Annexin Red Kit (Merck Millipore), according to the manufacturer's instructions. Briefly, cells were stained with annexin V conjugated with a sensitive dye CF647 (excitation laser: 642 nm, emission max: 670 nm) for 15 min at 37°C, washed in assay buffer, then stained with 7-AAD (excitation laser: 488 nm, emission max: 642 nm) for 5 min and analysed by Amnis FlowSight IS100 (Merck Millipore). Brightfield aspect ratio versus brightfield area plots were generated to identify single cells events, then 20,000 single-cell events for sample were acquired. Dot plots were obtained by plotting the fluorescence of annexin V (channel 11) versus fluorescence of 7-AAD (channel 5), resulting in four different populations: (1) healthy cells, annexin V(−), and 7-AAD(−); (2) necrotic cells, annexin V(+), and 7-AAD(+); (3) early apoptotic cells, annexin V(+), and 7-AAD(−); and (4) late apoptotic cells, annexin V(−), and 7-AAD(+). This technique has been used already by us in the study of the hyperthermia effects on dendritic cells [32] and applied to hAMSC : CFBE cocultures.

### 2.12. Statistical Analysis

Statistical significance of differences was evaluated by a two-tailed unpaired Student's *t*-test or ANOVA with Tukey's multiple comparison test. Data were analysed by using Prism 5 (GraphPad Software Inc., La Jolla, CA, USA). *p* values of less than 0.05 were considered significant.

## 3. Results

### 3.1. Basal Cx43 mRNA and Protein Expression

**Figure 1:**
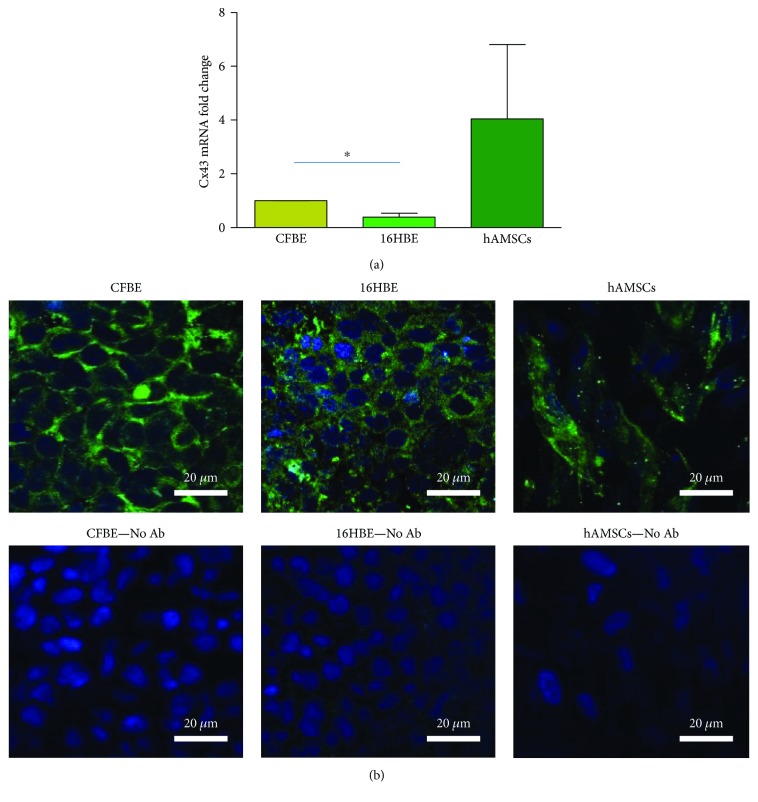
Basal expression of Cx43 mRNA and protein. (a) mRNA levels: CFBE, 16HBE, and hAMSCs were grown on Transwells for 6 days at confluence and then evaluated by real-time PCR. Values are relative to those of CFBE cells that were set at 1 and are shown as fold change. Results are shown as mean ± SD of three experiments, each carried out in triplicate. ^∗^ *p* < 0.05. (b) Cx43 protein expression and localisation. CFBE, 16HBE, and hAMSCs were grown on Transwells for 6 days at confluence and then stained with an Alexa Fluor 488-conjugated antibody directed against Cx43 (upper panels). As a control for background levels, cells were not treated with the primary antibody (lower panels). Original magnification: 60x. Scale bar: 20 *μ*m.

In order to investigate whether Cx43 could be a suitable target to be downregulated by siRNA, we studied its mRNA levels in CFBE and hASMCs, when grown on Transwells for 6 days. For comparison purposes, also 16HBE cells, expressing wild-type CFTR, were included in the analysis. As shown in Figure 1(a), CFBE displayed higher levels of Cx43 mRNA than 16HBE cells (*p* < 0.05 by unpaired Student's *t*-test). Although Cx43 mRNA levels have the tendency to be higher in hAMSCs than in both epithelial cell lines, these differences were not statistically significant.

When we analysed the expression and localisation of the Cx43 protein by epifluorescence and confocal microscopy in the three cell types grown on Transwells for 6 days, the XY images showed a similar signal in CFBE and 16HBE cells, localised at the cell borders (Figure 1(b)). hAMSCs were characterized by Cx43 localisation both at the cell membrane and within the cytoplasm (Figure 1(b)), a pattern previously found by Valiunas et al. [33] in MSCs obtained from human bone marrow that is considered the gold standard in the field of MSCs.

### 3.2. Downregulation of Cx43 by siRNA

The effect of siRNA on the target mRNA is exerted in the cytosol through the RNA-induced silencing complex (RISC) [34]. Thus, we studied the uptake and internalisation of siRNA by a cytofluorimetric assay, which allows to distinguish between the extracellular and intracellular siRNA molecules [35]. Since the target of downregulation studies is the coculture of hAMSCs and CFBE cells, cocultures at 1 : 5 hAMSC : CFBE ratio was used in this assay. After 24 h of incubation with fluorochrome-conjugated siRNA, cells were treated or not with trypan blue before analysis. Trypan blue quenches extracellular fluorescence allowing to determine intracellular signal only [35]; therefore, whole cell-associated fluorescence is detected in cells not treated with trypan blue, while internalised fluorescence is revealed in the treated ones. As reported in the Supplementary Figure 1, trypan blue-treated cells show almost 100% of positivity and this percentage is not different from that of cells evaluated in the absence of trypan blue pretreatment, thereby indicating that internalisation has occurred in all cells.

**Figure 2:**
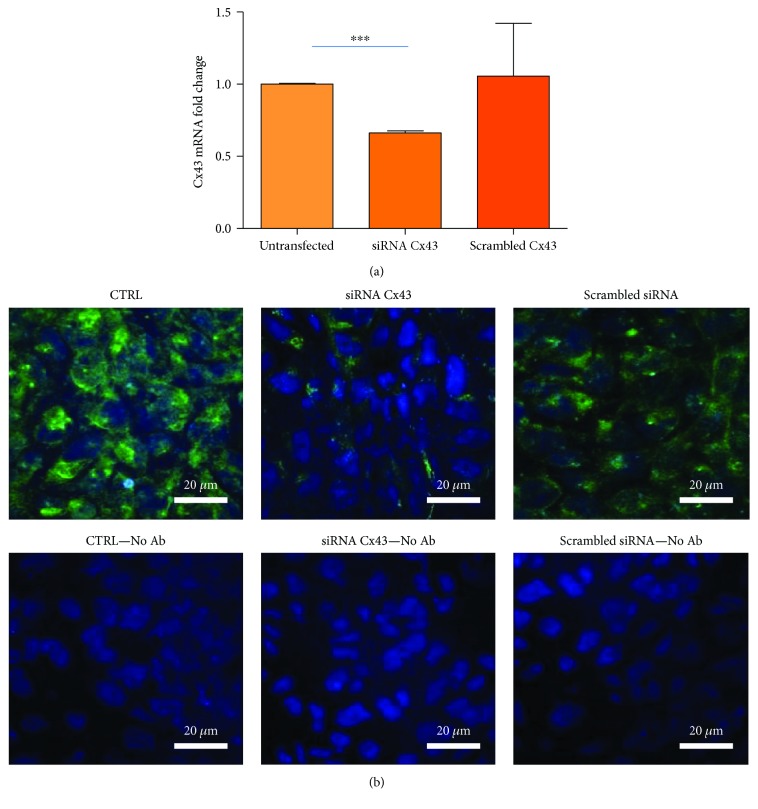
Downregulation of Cx43 mRNA and protein induced by a specific siRNA in cocultures. (a) Cx43 mRNA levels. 1 : 5 hAMSC : CFBE cocultures were transfected with either Cx43 siRNA or scrambled siRNA for 6 days. In parallel, other cocultures were not transfected and Cx43 mRNA levels obtained from these cocultures were set at 1. Data are shown as mean ± SD of three experiments, each carried out in duplicate. ^∗∗∗^ *p* < 0.0001 versus untransfected cocultures. (b) Cx43 protein levels and localisation. With the same experimental setting described in (a), cocultures were evaluated for Cx43 protein by confocal microscopy. The upper panels refer to cocultures incubated with the Alexa Fluor 488-conjugated antibody directed against Cx43, while the lower panels to cocultures in the absence of the primary antibody. Original magnification: 60x. Scale bar: 20 *μ*m.

siRNA internalisation was investigated in terms of efficacy as downregulation of Cx43 mRNA and protein, as well as the inhibition of functional GJs. As shown in Figure 2(a), only cocultures treated with the Cx43 siRNA show a significant decrease in Cx43 mRNA in respect to both untreated cocultures and coculture transfected with a scrambled siRNA, demonstrating the specific siRNA effect.

Confocal microscopy revealed that Cx43 is expressed both at the level of the plasma membrane and cytoplasm (Figure 2(b)). The treatment with the Cx43siRNA strongly downregulated the protein at both cellular localisations, while scrambled siRNA transfection exerted a small decrease on Cx43 at the plasma membrane level (Figure 2(b)).

**Figure 3:**
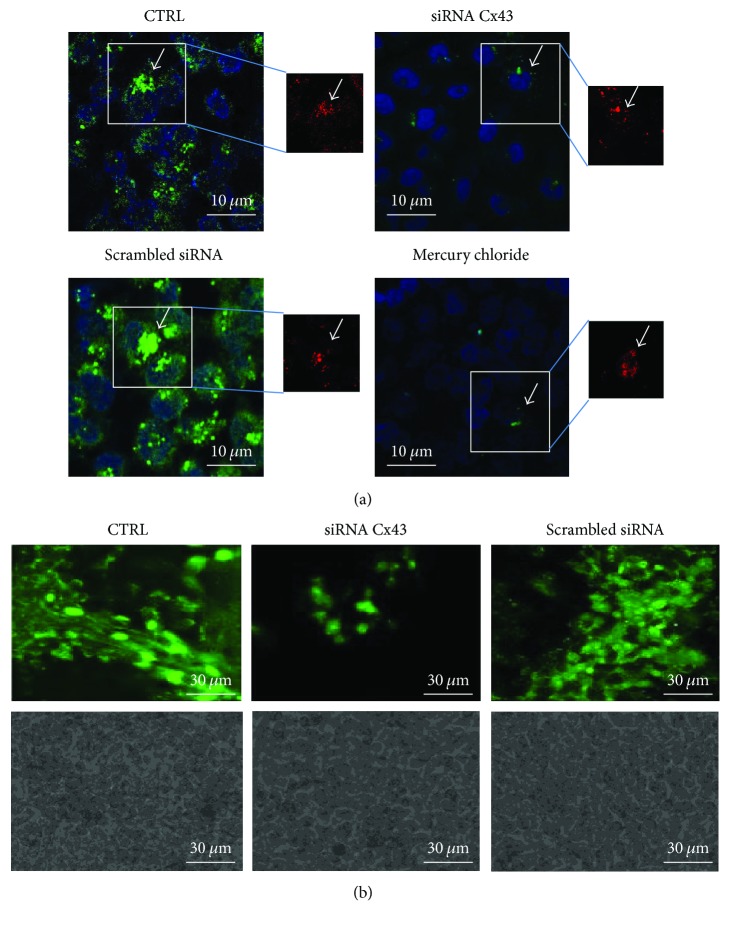
Effect of the siRNA Cx43 on Lucifer yellow (LY) diffusion in cocultures. (a) Mixed hAMSC-CFBE cells were transfected with siRNA against Cx43 or the scrambled siRNA or left untreated, cultured on glass slides, and evaluated for LY diffusion after 6 days by confocal microscopy (XY images). As a positive control of GJIC inhibition, 10 nM mercury (II) chloride was added to cocultures on the fifth day. Original magnification: 60x and 2.5x zooming. The inset panels show the injected cell indicated by the white arrow. The same cell with the dextran in red is shown in the side panels. Scale bar: 10 *μ*m. (b) Mixed hAMSC-CFBE cells were transfected with siRNA against Cx43 or the scrambled siRNA or left untreated, cultured on Transwells, and evaluated for LY diffusion after 6 days by confocal microscopy (XY images). Original magnification: 60x. Scale bar: 30 *μ*m.

In order to study the formation of functional gap junctions, we used the microinjection of Lucifer yellow (LY), a fluorescent dye which diffuses among cells through GJs [30]. Texas Red-conjugated dextran was also used to highlight the injected cells, since it does not pass through GJs. Cocultures grown on slides displayed a broad diffusion of LY from the injection point, while the treatment with the active siRNA led to a drastic reduction in cell-to-cell diffusion (Figure 3(a)). Cocultures treated with the scrambled siRNA resembled those untreated. As a positive control of inhibition of GJCs, HgCl_2_ (at a subcytotoxic concentration as low as 10 nM) was shown to cause a significant reduction in cell-to-cell coupling after a 24 h of incubation. Under these experimental conditions, autofluorescence levels were negligible in both green and red channels (Supplementary Figure 2). siRNA efficiency was also tested in cocultures seeded onto Transwells, finding that LY diffusion was almost abolished by the Cx43 siRNA and not by control siRNA (Figure 3(b)). Altogether, these data show the important role of Cx43 in the GJ formation and function in cocultures.

### 3.3. Reduction in CFTR Protein Expression and Function by siRNA Downregulation

**Figure 4:**
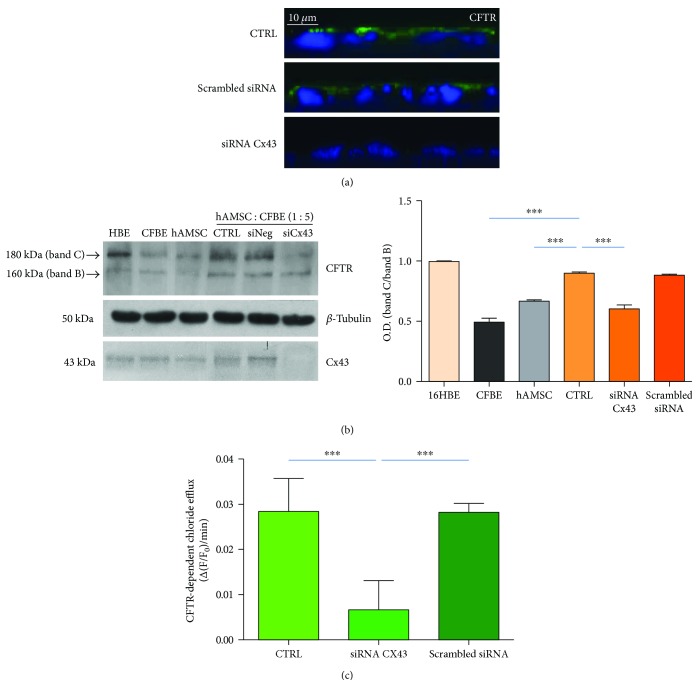
Effect of Cx43 siRNA on CFTR protein localisation, expression, and function. (a) Confocal microscopy of cocultures after 6 days from the transfection with either siRNA against CX43 or scrambled siRNA. Cocultures were stained with an anti-CFTR primary antibody followed by an Alexa Fluor 488-conjugated secondary antibody (green) and DAPI (blue). XZ images are shown. Original magnification: 60x. Scale bar: 10 *μ*m. (b) Representative Western blot of a typical experiment analysed using anti-human CFTR C-terminus antibody (dilution 1 : 500), anti-Cx43 (dilution 1 : 500), anti *β*-tubulin (dilution 1 : 1000). *β*-Tubulin was used to normalise protein loading. The histogram summarises the relative change in the ratio between band C and band B in the various conditions compared to the CFTR ratio in the 16HBE set as 1. Results represent means ± SEM of three experiments. ^∗∗∗^ *p* < 0.0001. (c) CFTR-dependent chloride efflux analysed under the same experimental conditions. Data are shown as mean ± SD of three experiments. ^∗∗∗^ *p* < 0.0001.

Having demonstrated that a siRNA against Cx43 is able to block cell-to-cell communication via gap junctions, we next evaluated the effect of this siRNA on CFTR expression and function. In order to demonstrate that the GJ-mediated intercellular communication is involved in the rescue of expression, localisation, and function of CFTR protein, hAMSC-CFBE mixed cells (at the 1 : 5 ratio) were transfected with either Cx43 siRNA or scrambled siRNA or left untreated and then cocultured on Transwells. As shown in Figure 4(a), confocal analysis of the CFTR protein distribution showed that in untransfected cocultures as well as in cocultures transfected with the scrambled siRNA, CFTR was localised at the apical membrane, whereas Cx43 silencing resulted in the disappearance of CFTR from this cellular region.

CFTR is a glycoprotein that exits from the endoplasmic reticulum (ER) as 160 kDa immature form (band B) and then is further glycosylated to a 180 kDa mature form (band C), which is finally transported to the plasma membrane. We confirmed confocal microscopy results by Western blotting analysis performed in 16HBE (endogenously expressing wt CFTR and therefore used as a reference to the location of band C and band B of wt CFTR), CFBE, hAMSCs, and cocultures transfected with either siRNA for Cx43 or scrambled siRNA grown on Transwells.  Figure 4(b) shows a typical Western blot; in 16HBE cells, the mature band C of CFTR was strongly expressed; on the contrary, in the CFBE cells, F508del CFTR was almost completely absent as well as in hAMSCs. As we previously showed [18], 1 : 5 hAMSC : CFBE cocultures displayed an increase in the mature band C, indicating the rescue of CFTR maturation defect occurring in CFBE cells. Interestingly, band C almost completely disappeared when cocultures were transfected with siRNA for Cx43 but not with a scrambled siRNA, whereas band B intensity did not change (Figure 4(b)). Densitometric analysis revealed that Cx43 silencing significantly reduced CFTR protein expression (shown as band C/band B ratio) by 33% in respect to nontransfected (CTRL) or scrambled siRNA-treated cells (Figure 4(b)).

To determine whether the rescue of CFTR-dependent chloride secretion in cocultures is affected by Cx43-dependent permeability, cells were transfected with the siRNA against Cx43 or left untreated and then analysed for apical CFTR-dependent chloride efflux. The function of the CFTR protein was evaluated by spectrofluorimetric measurements of chloride efflux detecting the change in fluorescence (Δ(F/F_0_)/min) of the chloride sensitive dye MQAE as previously reported [7]. As shown in Figure 4(c), the CFTR-dependent chloride efflux was similar in untreated cocultures and in those transfected with the scrambled siRNA. Importantly, the active siRNA transfection resulted in a significant reduction of chloride efflux in comparison to the other two conditions, suggesting a central role of GJs in the cross-talk between hAMSCs and CFBE cells and the rescue of CFTR protein expression and function.

### 3.4. Cx43 Downregulation Determines a Decrease in Transepithelial Resistance

**Figure 5:**
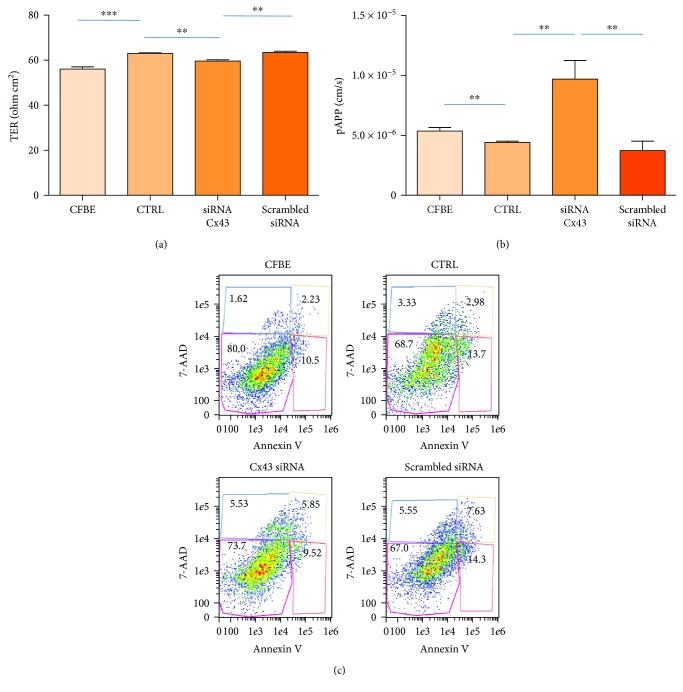
Effect of Cx43 siRNA on transepithelial resistance and cell viability. (a) Trans-epithelial resistance (TER; ohm cm^2^) was measured after 6 days from the transfection with either siRNA against Cx43 or scrambled siRNA. Untreated cocultures (CTRL) and CFBE cells cultured on Transwells were also analysed. Data were obtained from three experiments, each carried out in quadruplicate. Results are shown as mean ± SEM. ^∗∗^ *p* < 0.01; ^∗∗∗^ *p* < 0.0001. (b) Paracellular permeability (pAPP; cm/s) was measured after 6 days in the same experimental conditions as in (a). Data were obtained from three experiments, each carried out in triplicate. Results are shown as mean ± SEM. ^∗∗^ *p* < 0.01. (c) Representative dot plot showing viable (7-AAD(−) and annexin V(−)), necrotic (7-AAD(+) and annexin V(+)), early apoptotic (7-AAD(−) and annexin V(+)), and late apoptotic (7-AAD(+) and annexin V(−)) cells in all conditions as in (a). One representative experiment is shown.

In CFBE cells, CFTR overexpression has been shown to increase both the CFTR trafficking to the plasma membrane and the transepithelial resistance (TER) while reducing the permeability to small molecules [36]. Therefore, we considered the epithelial resistance as a representative measurement of wt CFTR expression and transport to the apical membrane. We found that in comparison with the CFBE cells, the CFBE : hASMC cocultures, on the sixth day, displayed a significantly higher TER values than the CFBE cells (Figure 5(a)). As expected, Cx43 siRNA transfection of cocultures significantly reduced TER, while the scrambled siRNA transfection was ineffective with respect to control cocultures. To understand whether the changes in TER could be biologically relevant, we studied the paracellular permeability to a FITC-conjugated dextran (10 s; 10,000 Da). As shown in Figure 5(b), the pAPP was significantly lower in control CFBE : hAMSC cocultures, paralleling the increase in TER. Cx43 siRNA transfection of cocultures leads to a significant increase in pAPP as compared with both controls and cocultures transfected with the scrambled siRNA.

In order to see whether TER alterations could be due to a marked modification of viability, we analysed apoptosis and necrosis by a cytofluorimetric assay (Figure 5(c)). Although there was a significant decrease in cell viability in control cocultures and cocultures transfected with scrambled siRNA as compared with CFBE single cultures (Supplementary Table 1), the percentages of viable (7-AAD(−) and annexin V(−)), necrotic (7-AAD(+) and annexin V(+)), early apoptotic (7-AAD(−) and annexin V(+)), and late apoptotic (7-AAD(+) and annexin V(−)) cells were not different among control cocultures and cocultures transfected with either Cx43 or the scrambled siRNA. These results make unlikely that TER and pAPP alterations are determined by a change in cell viability.

## 4. Discussion

MSCs have been shown to produce a wealth of healthy effects on the respiratory tract in many pathological conditions, including acute and chronic lung diseases [18, 19, 37]. MSCs might provide the tissue microenvironment with paracrine mediators which act on the different cellular structural components of the lung and which can have cytoprotective effects, as well as anti-inflammatory and immunomodulatory capacities [38]. These therapeutic effects are believed to be given minimally by their transdifferentiation to epithelial cells [39]. Nevertheless, it has been shown that human lung-resident MSCs, when administered to the mouse lung, can establish GJ communications with lung alveolar and bronchial epithelial cells and persist for up to 6 months [40]. Therefore, it is likely that a combination of paracrine and GJ-mediated factors might contribute to the therapeutic effects of MSCs at the level of the respiratory mucosa.

This study underlines the relevance of GJs in the rescue of CFTR protein expression and function as a chloride channel in CFBE : hAMSCs cocultures. Notably, the siRNA directed against Cx43 strongly reduced band C but not band B of CFTR; these results correlate with the significant decrease in CFTR protein localisation at the level of the plasma membrane and in CFTR chloride channel activity. By using the same confocal microscopic and fluorimetric assays, we have previously shown that CFBE cells display a lack of CFTR protein apical localisation and function that correlated with defective accumulation of cAMP in the subcortical compartment and reduced subcortical levels of PKA activity [31]. These alterations would be worth to be studied in cocultures before and after Cx43 siRNA transfection. At the moment, we are not able to explain why siRNA Cx43 does not modify CFTR-B expression, whereas it reduces CFTR-C expression. We can only hypothesise that the absence of modification in core-glycosylated band B could be consequent to the fact that Cx43 silencing induces an alteration of the ubiquitin proteasome pathway, which has been demonstrated to regulate CFTR degradation [41], or leads to its stabilization in the ER. Further studies are required to clarify these hypotheses.

These findings may be relevant to understand the mechanism of rescue of CFTR-dependent chloride efflux in the nose and rectum of CF mice with the administration of hematopoietic stem cells [42] or mesenchymal stem cells [43] derived from the bone marrow which resulted in modest or rare appearance of stem cell-derived epithelial cells in the lung, suggesting that few stem cells homed to the CF lung may trigger correction of CFTR defects in ion transport via cell-to-cell communications. The type of intercellular junctions that can be formed during cocultures is yet to be elucidated. Islam and colleagues found that Cx43-GJs mediated both cytoplasmic and mitochondrial transfer from bone marrow MSCs to alveolar epithelial cells [44]. On the other hand, it has been recently demonstrated that MSCs, both resident in the lung and bone marrow-derived, employ non-Cx43-GJs in the transfer of cytoplasmic content and mitochondria to bronchial epithelial cells in vitro [45]. Thus, it is possible that hAMSCs, once acquired an epithelial phenotype, may interact with bronchial epithelial cells through different types of GJs.

In the airways, GJs regulate Ca^2+^ wave propagation [46] and ciliary beat frequency [47] and coordinated secretion of airway surface fluid [27] and mucins [48], as well as innate immune functions of the epithelial layer [48–51]. Our previously published data [20] strongly suggested that intercellular communication is involved in the rescue of CF-associated basic defects by hAMSCs in coculture with CFBE cells. The mechanistic relationship between GJs and CFTR is not known at the moment; Chanson and colleagues [52] have suggested that CFTR may influence the function of GJs in a variety of cells, including pancreatic duct and airway epithelial cells. This study demonstrates that the interaction of CFTR with other intracellular proteins may provide a molecular mechanism for the regulation of GJ channels. Interestingly, our data indicate that the presence of functional GJs may allow the cell-to-cell transport of mediators that could rescue the correct localisation of the CFTR protein and its function as a chloride channel. One hypothesis is that interference with GJ function may affect chloride efflux, as chloride from adjacent CF cells fails to diffuse through gap junctions into hAMSCs where CFTR functions and secretes chloride normally. Alternatively, transfer of miRNAs could be involved. At the moment, several miRNAs expressed in primary human epithelial cells which directly repress CFTR expression by binding to the 3′ UTR of the transcript have been identified [53–56]. On the other hand, in polarized Calu-3 epithelia, Ramachandran et al. [55] found that miR-138 increased CFTR mRNA and protein levels as well as CFTR-mediated Cl^−^ conductance.

In this study, we found that siRNA downregulation of Cx43 brought to a decrease in TER and a corresponding increase in paracellular permeability in the absence of overt cytotoxicity or induction of apoptosis. Notably, we recently used the same cytofluorimetric assay to show that dendritic cells undergo necrosis and apoptosis following their exposure to 39°C [32], suggesting that our transfection conditions do not cause any harm to cocultures of hAMSC with airway epithelial cells. These results are in agreement with our previous results showing that hAMSC : CFBE cocultures displayed a corrected CFTR protein in association with an increase in ZO-1 localisation at the intercellular borders [20]. Thereby, Cx43 siRNA, abrogating CFTR rescue on the plasma membrane, may alter the tightness of the airway epithelium by an indirect mechanism. This hypothesis fits with the indications that CFTR expression may influence the epithelium tightness. LeSimple and colleagues, in fact, found that overexpression of CFTR in CFBE cells increased TER and reduced mannitol permeability [36]. In keeping with these results, Castellani et al. [8] demonstrated that overexpression of CFTR or its interacting protein NHERF1 in CFBE cell monolayers induced the reorganisation of TJ proteins at the level of intercellular junctions and reduced the paracellular permeability to dextrans. Nevertheless, because the pathway of shuttling chloride to hAMSCs is disrupted, less chloride and consequently sodium and other cations may be transported less. Thus, TER is increased. TER is widely accepted as a measure of paracellular permeability and reflects the ionic conductance of the paracellular pathway in the epithelial monolayer [57]. However, recently, Molina et al. [28] have shown that CF airway epithelial cells (CuFi) display a lower TER, similar expression of TJ proteins (ZO-1 and ZO-2) on the plasma membrane and a Cx43 mistrafficking to the plasma membrane as compared with wild-type cells (NuLi), suggesting that a defect in gap junctions might influence TER. Overall, these results imply a role for CFTR expression and function in the correct assembly and function of tight and gap junctions.

Although derived from an in vitro study, these results may have consequences in the context of in vivo application of hAMSCs to the diseased CF lung. Indeed, the administration of hAMSCs may have a double outcome: (i) to replenish the stem cell niche in the airways with multipotent stem cells that could give rise to the differentiated cell types; and (ii) to contribute to the airway physiology by interacting via GJ with resident airway epithelial cells. What is unknown at the moment is if hAMSCs can be administered as such or should be instructed by signals derived from epithelial cells in vitro before their instillation to the lung conduits. Another point which deserves further studies is which should be the best way to administer MSCs to the CF lung [58]. Local (intratracheal) would be preferred to avoid systemic side effects, allocation of MSCs in the proximity of the injury site(s) and possibly of endogenous stem cell niches in the airways. However, the CF epithelium is covered in vivo by a dense mucus; therefore, mucolytics could be adjuvant in this scenario. A systemic administration (intravenous) of MSCs is also appealing due to the pulmonary “first pass” effect [59, 60]. Recent clinical data show that the systemic administration of MSCs to patients with various lung diseases has proved to be safe [61], and there are two ongoing phase I clinical trials in CF with MSC infusion (NCT02866721 and NCT03058068, see https://clinicaltrials.gov). However, whether inflammatory mediators (cytokines and chemokines) also increase the potential of MSCs to home, adhere to, and repair damaged airway epithelium is largely unknown. This is an issue which should be elucidated in future experiments to better understand the mechanism on how MSCs could be therapeutic for inflamed CF lung disease whether administered locally or systemically.

## 5. Conclusion

This study highlights the importance of GJs in the cross-talk occurring between mesenchymal stem cells and epithelial cells with CF leading to the rescue of a functional CFTR protein. It should be stressed that MSCs are able to secrete a host of paracrine factors with anti-inflammatory, immunomodulating, and anti-infective effects. As CF lung pathology is characterised by chronic inflammation and infections, future work should assess whether hAMSCs are useful in this context, besides the rescue of a mature and functional CFTR protein. Finally, in vivo studies on the role of direct coupling of hAMSCs and CF epithelial cells in the rescue of CFTR defects are deserved.

## Supplementary Materials

Supplementary Materials Supplementary Figure 1: internalisation of siRNA. Cocultures of hASMCs and CFBE (1 : 5 ratio) were transfected with a FITC-conjugated siRNA (see Materials and Methods). After 24 h, cells were treated or not with trypan blue (TB) prior to cytofluorimetric analysis. Results are shown as mean ± SD obtained from *n* = 2 experiments, each carried out in triplicate. Supplementary Figure 2: immunofluorescence controls. Cocultures of hASMCs and CFBE (1 : 5 ratio) were stained with DAPI and observed either at the DAPI channel (a), FITC channel (b), or TRITC channel (c). DAPI filter: 358 nm excitation, 461 nm emission; FITC filter: 490 nm excitation, 525 nm emission; TRITC filter: 532 nm excitation, 570 nm emission. Supplementary Table 1: effect of siRNA transfection on cell viability.
