# Supplementary material for: Gap Junctions Are Involved in the Rescue of CFTR-Dependent Chloride Efflux by Amniotic Mesenchymal Stem Cells in Coculture with Cystic Fibrosis CFBE41o- Cells
Source: Stem Cells Int. 2018 Jan 11;2018:1203717. doi: 10.1155/2018/1203717 (PMC5821953; doi:10.1155/2018/1203717)
Supplement: Supplementary Materials — Supplementary Figure 1: internalisation of siRNA. Cocultures of hASMCs and CFBE (1 : 5 ratio) were transfected with a FITC-conjugated siRNA (see Materials and Methods). After 24 h, cells were treated or not with trypan blue (TB) prior to cytofluorimetric analysis. Results are shown as mean ± SD obtained from n = 2 experiments, each carried out in triplicate. Supplementary Figure 2: immunofluorescence controls. Cocultures of hASMCs and CFBE (1 : 5 ratio) were stained with DAPI and observed either at the DAPI channel (a), FITC channel (b), or TRITC channel (c). DAPI filter: 358 nm excitation, 461 nm emission; FITC filter: 490 nm excitation, 525 nm emission; TRITC filter: 532 nm excitation, 570 nm emission. Supplementary Table 1: effect of siRNA transfection on cell viability. [file 1203717.f1.docx]

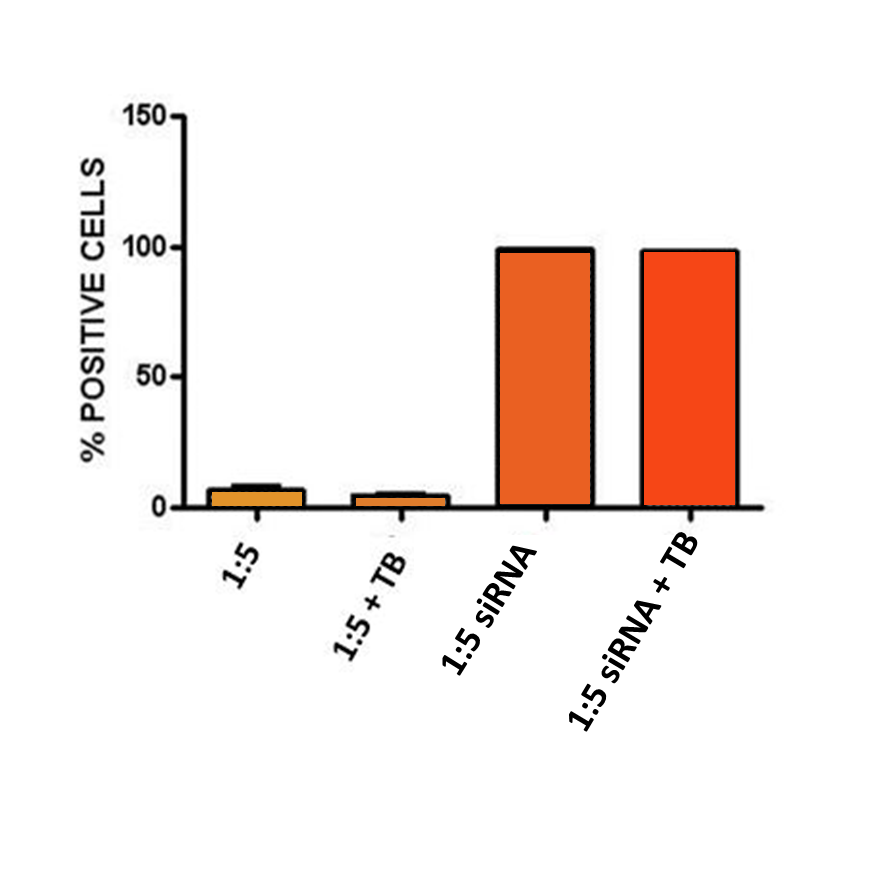


**Supplementary Figure 1.**Internalisation of siRNA. Co-cultures of hASMCs and CFBE (1:5 ratio) were transfected with a FITC-conjugated siRNA (see Materials and Methods). After 24 h, cells were treated or not with trypan blue (TB) prior to cytofluorimetricanalysis. Results are shown as mean±SD obtained from n=2 experiments, each carried out in triplicate.


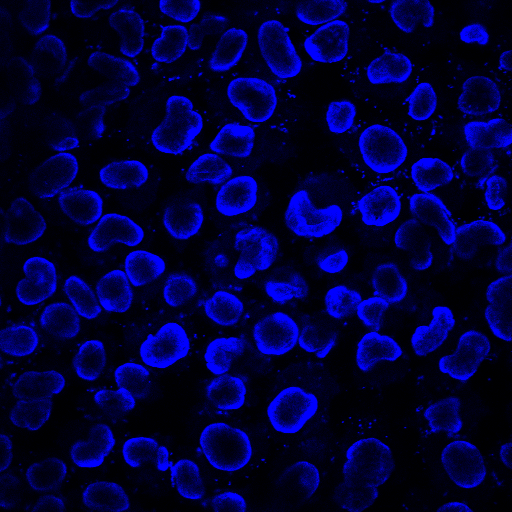

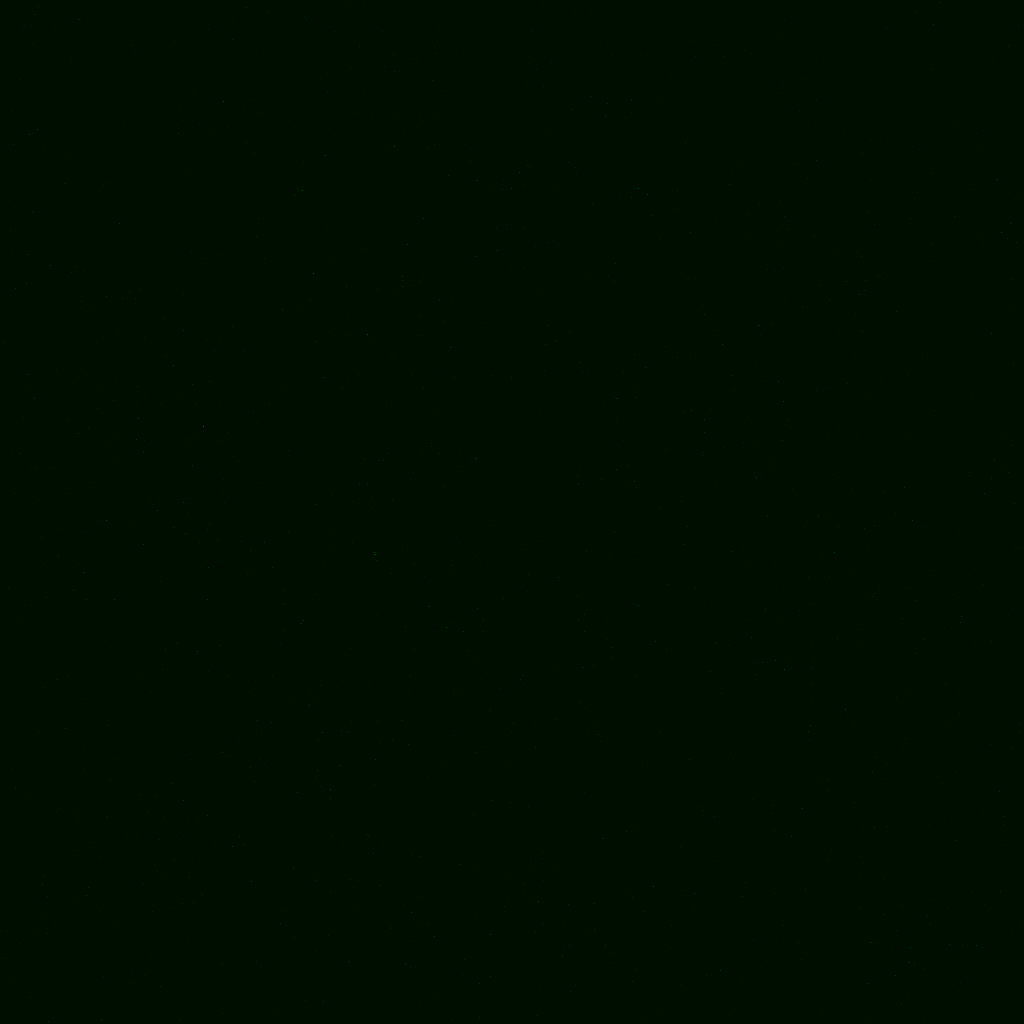

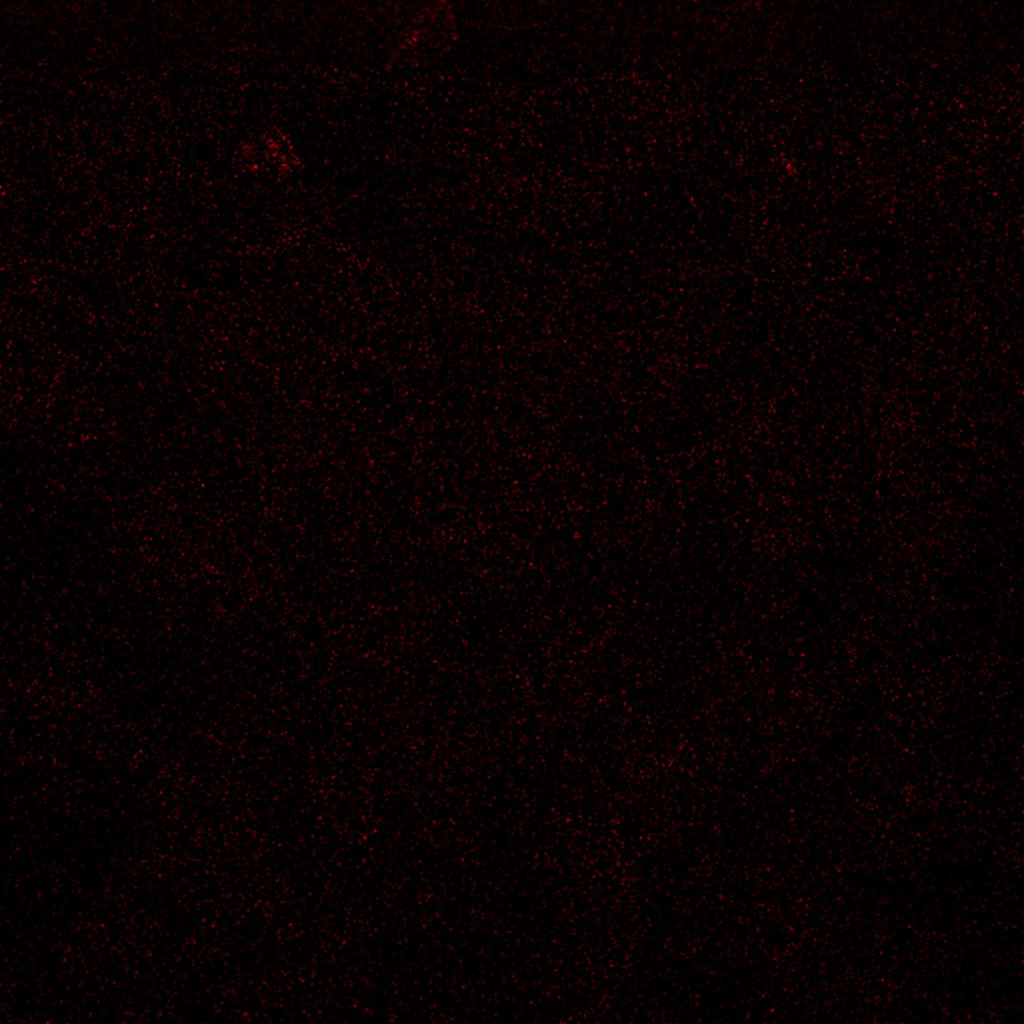


(a)

(b)

(c)

**Supplementary Figure 2**. Immunofluorescence controls.Co-cultures of hASMCs and CFBE (1:5 ratio) were stained with DAPI and observed either at the DAPI channel (**a**), FITC channel (**b**), or TRITC channel (**c**). DAPI filter: 358 nm excitation; 461 nm emission, FITC filter: 490 nm excitation; 525 nm emission, TRITC filter: 532 nm excitation; 570 nm emission.

Supplementary Table 1

Effect of siRNA transfection on cell viability.

| Single culture / co-culture | Viable | Necrotic | Early apoptotic | Late apoptotic |
| --- | --- | --- | --- | --- |
|  |  |  |  |  |
| CFBE | 82.9 (4.1) | 2.0 (0.2) | 7.1 (4.7) | 2.0 (0.5) |
| CTRL | 68.1 (0.7)* | 3.3 (0.5) | 9.5 (5.9) | 6.8 (4.9) |
| siRNA Cx43 | 73.6 (0.1) | 4.1 (2.4) | 7.5 (2.8) | 6.6 (1.6) |
| scrambled siRNA | 68.4 (2.0)* | 5.2 (3.3) | 9.6 (6.6) | 9.1 (5.1) |

Cell viability, necrosis and apoptosis were analysed after 6 days from the transfection with either siRNA against Cx43 or scrambled siRNA. Untreated co-cultures (CTRL) and single CFBE cells cultured on Transwells were also analysed. Viable (7AAD- Annexin V-), necrotic (7AAD+ Annexin V+), early apoptotic (7AAD- Annexin V+), and late apoptotic (7AAD+ Annexin V-) cells are represented in all conditions. Data were obtained from two experiments, each carried out in quadruplicate. Results are shown as mean (SD). *p<0.05 (ANOVA), CTRL and scrambled siRNA vs. CFBE.
